# Direct Enzymatic Route for the Preparation of Novel Enantiomerically Enriched Hydroxylated β-Amino Ester Stereoisomers

**DOI:** 10.3390/molecules15063998

**Published:** 2010-06-01

**Authors:** Enikő Forró, László Schönstein, Loránd Kiss, Alberto Vega-Peñaloza, Eusebio Juaristi, Ferenc Fülöp

**Affiliations:** 1 Institute of Pharmaceutical Chemistry, University of Szeged, H-6720 Szeged, Eötvös u. 6, Hungary; 2 Departamento de Química, Centro de Investigación y de Estudios Avanzados del Instituto Politécnico Nacional, Av. Instituto Politécnico Nacional 2508 Col. San Pedro Zacatenco, 07360 México, D.F. Apartado Postal 14-740, 07000, D.F., Mexico; 3 Research Group of Stereochemistry of the Hungarian Academy of Sciences, University of Szeged, H-6720 Szeged, Eötvös u. 6, Hungary

**Keywords:** β-amino ester, stereoisomer, enzyme, hydrolysis, hydroxylation, organic solvent, enantioselective

## Abstract

Enantiomerically enriched hydroxy-substituted *β*-amino esters have been synthesized through CAL-B-catalyzed enantioselective hydrolysis in organic media. Moderate to good enantiomeric excess values (*ee* ≥ 52%) were obtained when the CAL-B-catalyzed reactions were performed in *t-*BuOMe, at 60 ºC with 0.5 equiv. of added H_2_O as nucleophile.

## 1. Introduction

A number of cyclic *β*-amino acids exhibit biological activity (*e.g.* cispentacin [1,2] and Icofungipen [3,4]), and in addition they have also been used in peptide (modified activities and stabilities [5] and well-defined three-dimensional structures [6,7,8,9]), in heterocyclic [10,11,12,13] and combinatorial [14,15,16] chemistry, and in drug research [9,17,18]. Hydroxylated *β*-amino acids represent a valuable class of amino acids, due to their importance from both biological and chemical aspects. In recent years, acyclic hydroxylated *β*-amino acids have attracted much attention especially following their recognition as an important class of compounds in the design and synthesis of potential pharmaceutical drugs [e.g., Taxol® (paclitaxel) and its analogue Taxotère® (docetaxel)] [17,19,20,21]. Among the carbocyclic β-amino acids a number of hydroxylated β-amino acid derivatives present antibiotic (e.g., oryzoxymycin [22]) or antifungal activity, and are building blocks for pharmaceutically important natural substances such as fortamine, chryscandin, pentopyranamine, gougerotin and blasticidin [20,23].

Enzymatic methods for the preparation of several enantiomerically pure hydroxy-substituted carbocyclic *β*-amino acids or their derivatives have been published, but in those syntheses the enantioselective enzymatic step was performed for the preparation of starting *β*-amino acids [24,25,26,27]. In contrast, in this work the aim was to introduce the chirality on the last step on the synthetic sequence leading to hydroxy-substituted *β*-amino esters. Our earlier extensive investigations on enzyme-catalyzed enantioselective hydrolysis of both carbocyclic [28] and acyclic [29,30,31] *β*-amino esters suggested the possibility of direct lipase-catalyzed resolution of hydroxy-substituted *β*-amino esters (1R*,2S*,5S*)-(±)-**4**, (1S*,2S*,5R*)-(±)-**5**, (1S*,2S*,5S*)-(±)-**6** and (1S*,2S*,5R*)-(±)-**10**, through enantioselective enzymatic hydrolysis. 

## 2. Results and Discussion

### 2.1. Synthesis of hydroxy-substituted racemic β-amino esters (1R*,2S*,5S*)-(±)-4, (1S*,2S*,5S*)-(±)-5, (1S*,2S*,5S*)-(±)-6 and (1S*,2S*,5R*)-(±)-10

**Scheme 1:**
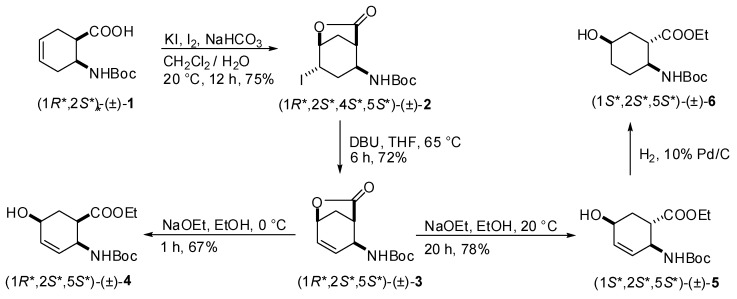
Synthesis of (1R*,2S*,5S*)-(±)-**4**, (1S*,2S*,5S*)-(±)-**5** and (1S*,2S*,5S*)-(±)-**6**.

The selective synthesis of the hydroxylated amino ester stereoisomers with a cyclohexene framework has been accomplished starting from *N*-Boc protected *cis*- or *trans*-2-aminocyclohex-3-enecarboxylic acid [32] and was based on stereoselective iodolactonization followed by dehydroiodination and lactone opening. Thus, amino acid (1*R*,2*S*)-(±)-**1** on treatment with KI/I_2_ in the presence of NaHCO_3_ gave regioselectively iodolactone (1R*,2S*,4S*,5S*)-(±)-**2**, which was then subjected to dehydroiodination with DBU under basic conditions, affording unsaturated lactone (1R*,2S*,5S*)-(±)-**3** (Scheme 1). The hydroxyl function in the cyclohexene skeleton was introduced by lactone opening of (1R*,2S*,5S*)-(±)-**3** with NaOEt. When the reaction was performed in EtOH at 0 °C for 1 h the *all-cis*-5-hydroxlated amino ester (1R*,2S*,5S*)-(±)-**4** was formed in good yield. By contrast, upon reaction with NaOEt at 20 °C for 20 h lactone (1R*,2S*,5S*)-(±)-**3** underwent epimerization at C-1 to give the hydroxylated amino ester stereoisomer (1S*,2S*,5S*)-(±)-**5** (Scheme 1). The desired saturated analog of (1S*,2S*,5S*)-(±)-**6 **was readily prepared in good yield by Pd catalyzed hydrogenation performed at room temperature and atmospheric pressure. 

**Scheme 2:**
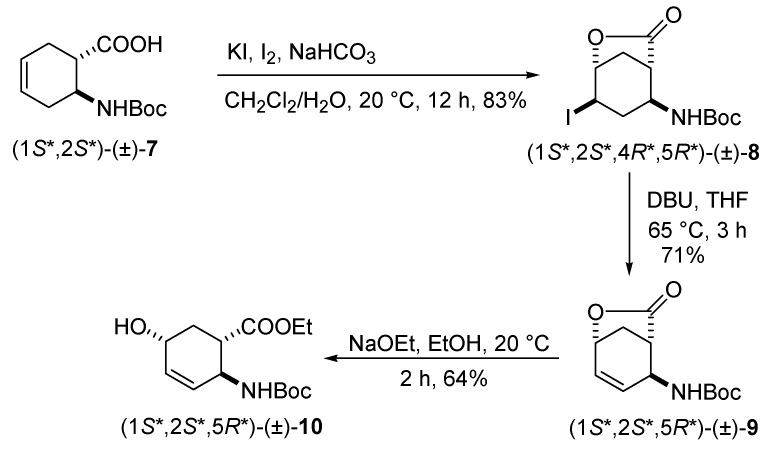
Synthesis of (1S*,2S*,5R*)-(±)-**10**.

Other hydroxylated stereoiomers of (1R*,2S*,5S*)-(±)-**4** and (1S*,2S*,5S*)-(±)-**5 **could be prepared from *N*-Boc-protected amino acid (1S*,2S*)-(±)-**7** when submitted to iodolatonization, dehydroiodination, followed by lactone ring-opening with NaOEt to furnish selectively hydroxylated amino ester stereoisomer (1S*,2S*,5R*)-(±)-**10 **(Scheme 2). 

### 2.2. Enzymatic hydrolysis of hydroxy-substituted β-amino esters (1S*,2R*,5R*)-(±)-4, (1S*,2S*,5S*)-(±)-5, (1S*,2S*,5S*)-(±)-6 and (1S*,2S*,5R*)-(±)-10

**Scheme 3:**
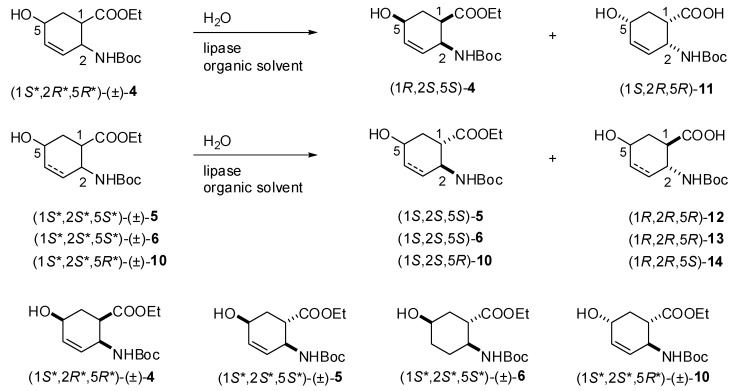
Enzymatic hydrolysis of (1S*,2R*,5R*)-(±)-**4**, (1S*,2S*,5S*)-(±)-**5**, (1S*,2S*,5S*)-(±)-**6** and (1S*,2S*,5R*)-(±)-**10**.

On the basis of our earlier results on the enantioselective hydrolyses of *β-*amino esters using CAL-B and lipase PS in an organic solvent [28,29,30,31] we carried out enzymatic screening experiments relating to the hydrolysis of (1S*,2S*,5R*)-(±)-**10** (Scheme 3) with 0.5 equiv. of H_2_O in *i-*Pr_2_O (Table 1, entries 1 and 11-15). Excepting lipase AK (*Pseudomonas flurescens*), which practically did not show any selectivity, all the other lipases tested [CAL-A (*Candida antarctica* lipase A), CAL-B (*Candida antarctica* lipase B), lipases PS (*Burkholderia cepacis*), AY (*Candida rugosa*) and PPL (porcine pancreatic lipase)] all showed catalytic activity, but only CAL-B catalyzed the reaction at 60 ºC with moderate enantioselectivity (*ee_substrate_* 48% after 48 h). As it was later confirmed, the majority of the antipodes of unreacted *β*-amino acid ethyl ester enantiomers were consumed in side-reactions (polymerization). 

In order to enhance the observed enantioselectivity, several solvents were tested in the CAL-B-catalyzed hydrolysis of (1S*,2S*,5R*)-(±)-**10** at 60 ºC (Table 1, entries 2-5). It could be appreciated that the reaction proceeded more slowly and less enantioselectively in *n*-hexane and 1,4-dioxane (entries 2 and 4), with better *E* and comparable reaction rate in toluene (entry 3) and with considerably higher *E* and higher reaction rate in *t-*BuOMe (entry 5) relative to *i*Pr_2_O (entry 1). 

**Table 1:** Conversion and enantioselectivity of enzymatic hydrolysis of (1S*,2S*,5R*)-(±)-**10**^a^.

Entry	Enzyme	Quantity of enzyme (mg mL^-1^)	Temperature (ºC.)	Solvent	Time (h)	*Conv*.^b^ (%)	*ee*_S_^c ^(%)	*ee*_P_^d ^(%)	*E* ^b^
1	CAL-B	30	60	*i*Pr_2_O	48	37	48	82	16
2	CAL-B	30	60	*n*-hexane	48	24	18	58	4
3	CAL-B	30	60	toluene	48	35	48	91	34
4	CAL-B	30	60	1,4-dioxane	48	25	16	48	3
5	CAL-B	30	60	*t-*BuOMe	48	45	75	91	48
6	CAL-B	30	50	*t-*BuOMe	48	35	47	86	21
7	CAL-B	30	40	*t-*BuOMe	48	36	46	81	15
8	CAL-B	30	70	*t-*BuOMe	40	35	44	82	16
9	CAL-B	50	60	*t-*BuOMe	40	40	53	81	16
10	CAL-B	75	60	*t-*BuOMe	40	42	60	83	20
11	Lipase PS^e^	30	45	*i*Pr_2_O	48	22	10	35	2
12	CAL-A^e^	30	45	*i*Pr_2_O	48	44	7	9	1
13	Lipase AY^e^	30	45	*i*Pr_2_O	48	11	2	16	1
14	Lipase AK^e^	30	45	*i*Pr_2_O	48	25	1	3	1
15	PPL	30	45	*i*Pr_2_O	48	20	5	20	2

^a ^0.05 M substrate; ^b ^Apparent values, calculated from *ee *for unreacted amino ester and *ee* for produced amino acid; ^c ^According to GC or HPLC (Experimental Section); ^d ^According to GC or HPLC after double derivatization (Experimental Section);^ e ^Contains 20% (w/w) of lipase adsorbed on Celite in the presence of sucrose.

**Figure 1:**
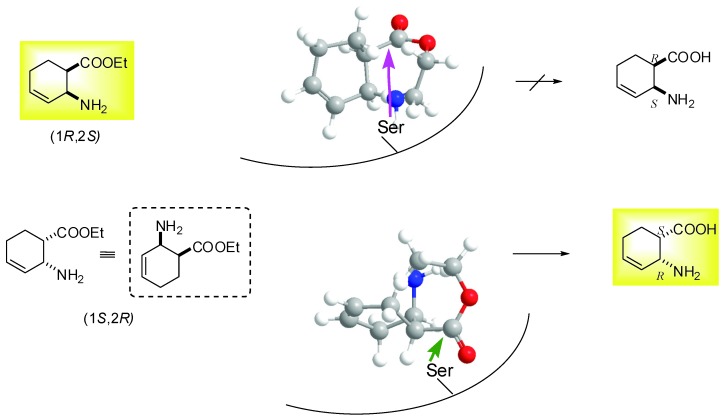
*S*-selective hydrolysis of ethyl *cis*-(2-aminocyclohex-3-ene)-1-carboxylate [28].

**Figure 2:**
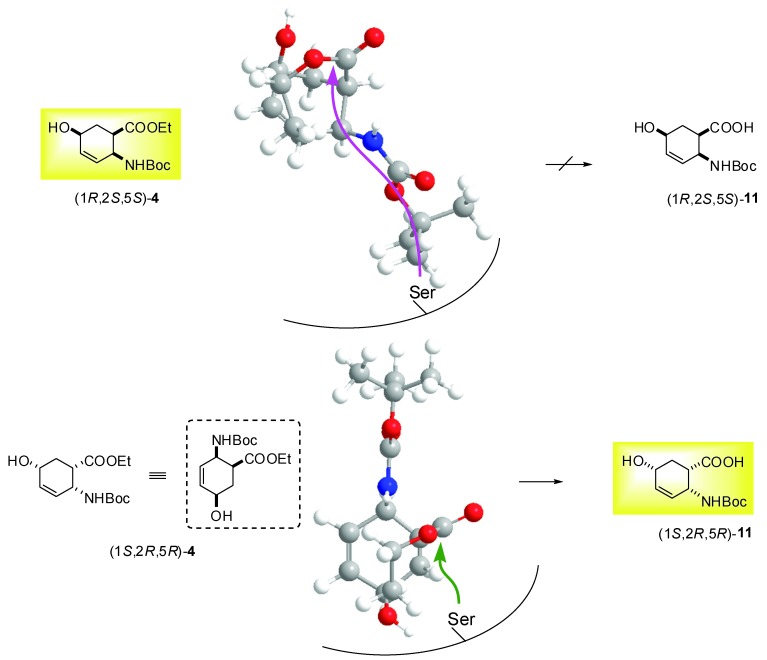
*S*-selective hydrolysis of (1S*,2R*,5R*)-(±)-**4**.

**Figure 3:**
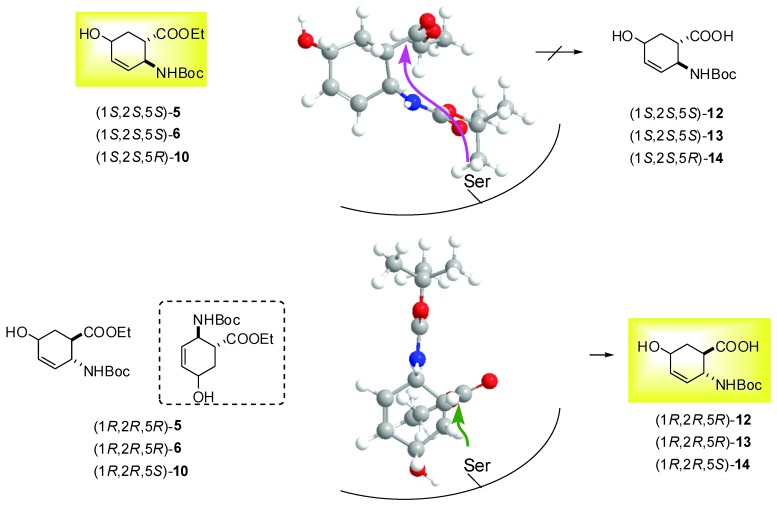
*R*-selective hydrolysis of (1S*,2S*,5S*)-(±)-**5**, (1S*,2S*,5S*)-(±)-**6** and (1S*,2S*,5R*)-(±)-**10**.

On the basis of these preliminary results, preparative-scale resolutions of (1S*,2R*,5R*)-(±)-**4**, (1S*,2S*,5S*)-(±)-**5**, (1S*,2S*,5S*)-(±)-**6** and (1S*,2S*,5R*)-(±)-**10** were performed in *t*-BuOMe at 60 ºC in the presence of CAL-B (30 mg mL^-1^) with 0.5 equiv. of H_2_O. The results are summarized in Table 2 and the Experimental Section. It can be noted, that the hydroxy-substituted *β*-amino acid enantiomers (**11**−**14**) are missing from the Table, as above observed, the *β*-amino acid ethyl esters (1*S*,2*S*,5*R*)-**4**, (1*R*,2*R*,5*R*)-**5**, (1*R*,2*R*,5*R*)-**6** and (1*R*,2*R*,5*S*)-**10** underwent polymerization (the side-product(s) were not characterized). As it was confirmed for the product obtained from (1*S*,2*S*,5*R*)-**4**, the mass spectrum shows two major peaks. The mass difference between these two peaks is 259.4 m/z. This corresponds to the mass of one Boc-protected saturated amino acid. Furthermore the MS/MS spectra of these two peaks show losses of Boc groups and Boc-protected saturated amino acid residues. These results suggest the polymerization of the amino acid derivative. A possible pathway is the enzyme catalyzed Michael addition. The NMR results support this view, because the signals of the protons of the double bonds and the OH groups are missing. Since CAL-B showed *S*-selectivity towards the earlier performed hydrolyses of *cis* and *R*-selectivity of *trans* carbocyclic *β*-amino esters [28], *S-*selectivity for hydrolysis of (1S*,2R*,5R*)-(±)-**4 **and *R*-selectivity for hydrolysis of (1S*,2S*,5S*)-(±)-**5**, (1S*,2S*,5S*)-(±)-**6** and (1S*,2S*,5R*)-(±)-**10** has been assumed. These lipase-catalysed reactions of hydroxy-substituted *β*-amino esters presumably proceeds in two stages: first, the Ser OH attacks the carbonyl group of ester function and forms the acyl-enzyme intermediate and second, deacylation of the enzyme in combination with side reactions (polymerization) results the products (*β*-amino acid being one of them). Even though modelling has not been used, knowledge of the active site structure of CAL-B, together with the stereostructures of hydroxy-substituted *β*-amino esters (CS Chem 3D 6.0) and a consideration of the results obtained for the CAL-B-catalysed hydrolysis of ethyl *cis*-(2-aminocyclohex-3-ene)-1-carboxylate (Figure 1), can be utilized to predict the interaction between the substrate and the acyl binding site. Figure 2 and Figure 3 illustrate the possible accommodations for the enantiomers in the apparent active site of CAL-B (only the Ser residue is indicated), which suggest the proposed selectivities, since the orientations (1*R*,2*S*,5*S*)-**4**, (1*S*,2*S*,5*S*)-**5**, (1*S*,2*S*,5*S*)-**6** and (1*S*,2*S*,5*R*)-**10** do not appear to be appropriate to ensure Ser OH attack.

**Table 2:** Lipase-catalyzed preparative-scale hydrolyses of (1S*,2R*,5R*)-(±)-**4**, (1S*,2S*,5S*)-(±)-**5**, (1S*,2S*,5S*)-(±)-**6** and (1S*,2S*,5R*)-(±)-**10**^a^.

Racemate	Time (days)	*Conv*.^b^ (%)	Unreacted enantiomer			
			Yield (%)	Isomer	*ee*^c ^(%)	[α]_D_^25 ^(EtOH)
(1 S*,2R*,5R*)-(±)-**4**	10	43	32	(1*R*,2*S*,5*S*)-**4**	68	+34 (*c* 0.19)
(1 S*,2S*,5S*)-(±)-**5**	10	41	34	(1*S*,2*S*,5*S*)-**5**	78	+16 (*c* 0.165)
(1 S*,2S*,5S*)-(±)-**6**	10	36	29	(1*S*,2*S*,5*S*)-**6**	54	-4 (*c* 0.24)
(1 S*,2S*,5R*)-(±)-**10**	7	43	41	(1*S*,2*S*,5*R*)-**10**	90	+100 (*c* 0.155)

^a^ 0.5 equiv. of H_2_O; CAL-B (30 mg mL^-1^) in *t-*BuOMe at 60 ºC; ^b ^Calculated using an internal standard (*n*-heptadecane, GC); ^c ^According to GC or HPLC (Experimental Section).

## 3. Experimental

### 3.1. Materials and methods

CAL-B (lipase B from *Candida antarctica*, produced by the submerged fermentation of a genetically modified *Aspergillus oryzae* microorganism and adsorbed on a macroporous resin) and porcine pancreatic lipase (PPL) were from Sigma-Aldrich. CAL-A (lipase A from *Candida antarctica*) was purchased from Roche Diagnostics Corporation. Lipase PS-IM (immobilized on diatomaceous earth) and PS–SD (*Burkholderia cepacia*) were kind gifts from Amano Enzyme Europe Ltd. Lipase AK (*Pseudomonas fluorescens*) was from Amano Pharmaceuticals, and lipase AY (*Candida rugosa*) was from Fluka. All the chemicals were purchased from Aldrich or Fluka. The solvents were of the highest analytical grade. Melting points were determined with a Kofler apparatus. NMR spectra were recorded on a Bruker DRX 400 spectrometer. Chemical shifts are given in ppm relative to TMS as internal standard, with CDCl_3_ or DMSO as solvent. Optical rotations were measured with a Perkin-Elmer 341 polarimeter. The mass spectra were recorded on a Finnigan MAT 95S spectrometer. Elemental analyses were performed with a Perkin-Elmer CHNS-2400 Ser II Elemental Analyzer. Compounds (1R*,2S*)-(±)-**1** and (1S*,2S*)-(±)-**7** were prepared according to our earlier method [32].

### 3.2. Typical small-scale enzymatic experiment

Racemic substrate (0.05 M solution) in an organic solvent (1 mL) was added to the lipase tested (30, 50 or 75 mg mL^-1^). Water (0.5 equiv.) was next added. The mixture was shaken at 40, 50, 60 or 70 °C. The progress of the reaction was followed by taking samples from the reaction mixture at intervals and analysing them by GC or HPLC. The *ee* values for the unreacted β-amino ester (1*S*,2*S*,5*R*)-**10 **and produced β-amino acid (1*R*,2*R*,5*S*)-**14 **enantiomers were determined by using HPLC equipped with Chiralpak IA 5 µ column (0.4 cm × 1 cm), after derivatization (Ac_2_O in the presence of 4-dimethyl-aminopyridine and pyridine) for (1*S*,2*S*,5*R*)-**10 **and double derivatization (CH_2_N_2_, Ac_2_O in the presence of 4-dimethylaminopyridine and pyridine) [33] for (1*R*,2*R*,5*S*)-**14 **[the mobile phase: *n*-hexane/2-propanol (90/10); flow rate 0.5 mL min^-1^; detection at 205 nm; retention times (min); (1*S*,2*S*,5*R*)-**10**: 28.96 (antipode: 24.96); (1*R*,2*R*,5*S*)-**14**: 28,06 (antipode: 36.05)]. The *ee* values for (1*R*,2*S*,5*S*)-**4**, (1*S*,2*S*,5*S*)-**5, **(1*S*,2*S*,5*S*)-**6, **(1*S*,2*R*,5*R*)-**11**, (1*R*,2*R*,5*R*)-**12 **and (1*R*,2*R*,5*R*)-**13, **were determined by using GC equipped with Chromopack Chiralsil-Dex CB column (25 m) after derivatization with CH_2_N_2_ [33] [190 °C; 140 kPa; retention times (min): (1*S*,2*S*,5*S*)-**5**: 12.67 (antipode: 13.28); (1*R*,2*R*,5*R*)-**12**: 11.80 (antipode: 11.30); (1*S*,2*S*,5*S*)-**6**: 13,29 (antipode: 14.51); (1*R*,2*R*,5*R*)-**13**: 12.87 (antipode: 12.38)], [170 °C; 140 kPa; retention times (min): (1*R*,2*S*,5*S*)-**4**: 27.06 (antipode: 28.12); (1*S*,2*R*,5*R*)-**11**: 14.47 (antipode: 13.76)]. 

### 3.3. Synthesis of iodolactones tert-butyl (1R*,2S*,4S*,5S*)-4-iodo-7-oxo-6-oxabicyclo[3.2.1]octan-2-ylcarbamate [(1R*,2S*,4S*,5S*)-(±)-2] and tert-butyl (1S*,2S*,4R*,5R*)-4-iodo-7-oxo-6-oxabicyclo-[3.2.1]octan-2-ylcarbamate [(1S*,2S*,4R*,5R*)-(±)-8] [32]

To a solution of *N*-Boc-protected amino acid [36 mmol (1R*,2S*)-(±)-**1** or (1S*,2S*)-(±)-**7**] in CH_2_Cl_2_ (200 mL), KI (5 equiv, 180 mmol), NaHCO_3_ (4 equiv, 144 mmol), H_2_O (200 mL) and I_2_ (2.1 equiv, 75 mmol) were added. After stirring for 12 h at room temperature, the mixture was taken up in CH_2_Cl_2_ (300 mL), and washed with saturated aqueous Na_2_SO_3_ solution. The organic layer was then dried (Na_2_SO_4_) and concentrated under reduced pressure and resulted (1R*,2S*,4S*,5S*)-(±)-**2** [white solid; yield: 75%; mp 180–183 °C (*n*-hexane); (R_f_ = 0.7, *n*-hexane-EtOAc 2:1)] or (1S*,2S*,4R*,5R*)-(±)-**8** [white solid; yield: 83%; mp 128–130 °C (*n*-hexane); (R_f_ = 0.65, *n*-hexane-EtOAc 2:1)]. 

^1^H-NMR (400 MHz, CDCl_3_) for (1R*,2S*,4S*,5S*)-(±)-**2**: δ = 1.48 (s, 9H, *t*Bu), 2.13–2.23 (m, 1H, CH_2_), 2.45–2.53 (m, 2H, CH_2_), 2.79–2.90 (m, 2H, H-1 and CH_2_), 4.16–4.22 (m, 1H, H-4), 4.48–4.53 (m, 1H, H-2), 4.78 (brs, 1H, N-H), 4.80–4.85 (m, 1H, H-5); ^13^C-NMR (100 MHz, CDCl_3_): δ = 20.8, 28.7, 33.9, 36.9, 44.9, 46.1, 80.1, 82.0, 156.8, 172.0. 

^1^H-NMR (400 MHz, CDCl_3_) for (1S*,2S*,4R*,5R*)-(±)-**8**: δ = 1.50 (s, 9H, *t*Bu), 2.23–2.30 (m, 1H, CH_2_), 2.33–2.43 (m, 1H, CH_2_), 2.87–3.07 (m, 3H, H-1 and CH_2_), 4.22–4.25 (m, 1H, H-4), 4.38–4.43 (m, 1H, H-2), 4.88–4.93 (m, 1H, H-5), 5.38 (brs, 1H, N-H); ^13^C-NMR (100 MHz, CDCl_3_): δ = 20.1, 28.5, 29.1, 33.9, 43.0, 46.6, 79.4, 81.7, 177.0, 179.0. 

### 3.4. Dehydroiodination reactions to obtain tert-butyl (1R*,2S*,5S*)-7-oxo-6-oxabicyclo[3.2.1]oct-3-en-2-ylcarbamate [(1R*,2S*,5S*)-(±)-3] and tert-butyl (1S*,2S*,5R*)-7-oxo-6-oxabicyclo[3.2.1]oct-3-en-2-ylcarbamate [(1S*,2S*,5R*)-(±)-9]

To a solution of iodolactone [12 mmol (1R*,2S*,4S*,5S*)-(±)-**2 **or (1S*,2S*,4R*,5R*)-(±)-**8**] in THF (60 mL) DBU (2.1 equiv, 25.2 mmol) was added and the mixture was stirred at 65 °C for 4 h. Then the solution was concentrated under reduced pressure and the residue was taken up in EtOAc (140 mL). The organic layer was washed with H_2_O (3 x 70 mL), dried (Na_2_SO_4_) and concentrated under vacuum. The residue was crystallized from *n*-hexane-EtOAc and resulted (1R*,2S*,5S*)-(±)-**3 **[white solid; yield: 72%; mp 148–149 °C (*n*-hexane-EtOAc); (R_f_ = 0.6, *n*-hexane-EtOAc 2:1)] or (1S*,2S*,5R*)-(±)-**9** [white solid; yield: 71%; mp 149–151 °C (*n*-hexane); (R_f_ = 0.6, *n*-hexane-EtOAc 2:1)]. 

^1^H-NMR (400 MHz, CDCl_3_) for (1R*,2S*,5S*)-(±)-**3**: δ = 1.48 (s, 9H, *t*Bu), 2.16–2.22 (m, 1H, CH_2_), 2.54–2.63 (m, 1H, CH_2_), 3.01–3.06 (m, 1H, H-1), 4.66–4.72 (m, 1H, H-2), 4.48–4.53 (m, 1H, H-2), 4.81–4.85 (m, 1H-5), 4.90 (brs, 1H, N-H), 5.78–5.82 (m, 1H, H-3), 6.30–6.37 (m, 1H, H-4); ^13^C-NMR (100 MHz, CDCl_3_): δ = 28.7, 37.1, 44.1, 48.9, 73.5, 80.6, 131.1, 132.1, 158.8, 172.4. 

^1^H-NMR (400 MHz, DMSO) for (1S*,2S*,5R*)-(±)-**9**: δ = 1.42 (s, 9H, *t*Bu), 2.28–2.36 (m, 2H, CH_2_), 2.69–2.75 (m, 1H, H-1), 4.20–4.28 (m, 1H, H-2), 4.71–4.76 (m, 1H, H-5), 5.59–5.65 (m, 1H, H-3), 6.40–6.46 (m, 1H, H-4), 7.48 (brs, 1H, N-H).^ 13^C-NMR (100 MHz, DMSO): 29.1, 31.5, 44.4, 47.6, 73.9, 79.4, 131.1, 132.9, 155.7, 178.2. 

### 3.5. Lactone ring opening reaction to obtain ethyl (1R*,2S*,5S*)-2-(tert-butoxycarbonylamino)-5-hydroxycyclohex-3-enecarboxylate [(1R*,2S*,5S*)-(±)-4], ethyl (1S*,2S*,5S*)-2-(tert-butoxycarbonyl-amino)-5-hydroxycyclohex-3-enecarboxylate [(1S*,2S*,5S*)-(±)-5] and ethyl (1S*,2S*,5R*)-2-(tert-butoxycarbonylamino)-5-hydroxycyclohex-3-enecarboxylate [(1S*,2S*,5R*)-(±)-10]

To a solution of unsaturated lactone [4 mmol of (1R*,2S*,5S*)-(±)-**3 **or (1S*,2S*,5R*)-(±)-**9**)] in anhydrous EtOH (20 mL) NaOEt (1.2 equiv, 4.8 mmol) was added at 0 °C and the reaction mixture was further stirred at the temperature and time indicated. Then the EtOH was removed under reduced pressure at 40 °C and the residue was taken up in EtOAc (50 mL). The organic layer was washed with H_2_O (3 × 25 mL), dried (Na_2_SO_4_) and concentrated under reduced pressure. The crude residue was crystallized from *n*-hexane-EtOAc to give the desired product: 

(1R*,2S*,5S*)-(±)-**4**: white solid; yield: 67%; mp 88–90 °C (*n*-hexane-EtOAc); (R_f_ = 0.4, *n*-hexane-EtOAc 1:1). ^1^H-NMR (400 MHz, CDCl_3_): δ = 1.26 (t, 3H, CH_3_), 1.42 (s, 9H, *t*Bu), 1.88–1.65 (m, 1H, CH_2_), 2.09–2.17 (m, 1H, CH_2_), 2.84–2.91 (m, 1H, H-1), 4.08–4.18 (m, 3H, OCH_2_ and H-2), 4.46–4.50 (m, 1H, H-5), 4.90 (brs, 1H, N-H), 5.67–5.71 (m, 1H, H-3), 5.82–5.88 (m, 1H, H-4); ^13^C-NMR (100 MHz, CDCl_3_): δ = 14.8, 29.0, 29.7, 42.9, 45.8, 61.6, 66.2, 78.6, 126.9, 125.9, 155.6, 172.8. 

(1S*,2S*,5S*)-(±)-**5**: white solid; yield: 78%; mp 107–108 °C (*n*-hexane-EtOAc); (R_f_ = 0.35, *n*-hexane-EtOAc 1:1). ^1^H-NMR (400 MHz, DMSO): δ = 1.18 (t, 3H, CH_3_), 1.37 (s, 9H, *t*Bu), 1.72–1.80 (m, 2H, CH_2_), 2.67–2.70 (m, 1H, H-1), 4.00–4.15 (m, 4H, OCH_2_, H-2 and H-5), 4.86 (brs, 1H, O-H), 5.46–5.52 (m, 1H, H-3), 5.68–5.73 (m, 1H, H-4), 6.68 (brs, 1H, N-H); ^13^C-NMR (100 MHz, DMSO): δ = 14.8, 29.1, 34.3, 41.8, 49.3, 60.8, 61.6, 78.6, 131.0, 131.4, 156.0, 174.6. 

(1S*,2S*,5R*)-(±)-**10**: white solid; yield: 64%; mp 130–133 °C (*n*-hexane-EtOAc); (R_f_ = 0.4, *n*-hexane-EtOAc 1:1). ^1^H-NMR (400 MHz, DMSO): δ = 1.17 (t, 3H, CH_3_, *J* = 7.15 Hz), 1.36 (s, 9H, *t*Bu), 1.46–1.53 (m, 1H, CH_2_), 1.97–2.07 (m, 1H, CH_2_), 3.96–4.11 (m, 3H, OCH_2_ and H-2), 4.18–4.25 (m, 1H, H-5), 4.90 (brs, 1H, O-H), 5.35–5.40 (m, 1H, H-3), 5.59–5.63 (m, 1H, H-4), 6.93 (brs, 1H, N-H); ^13^C-NMR (100 MHz, DMSO): δ = 14.9, 29.1, 36.1, 45.9, 49.6, 60.9, 65.6, 78.6, 129.9, 134.3, 155.9, 173.8. 

### 3.6. Preparation of ethyl (1S*,2S*,5S*)-2-(tert-butoxycarbonylamino)-5-hydroxycyclohexane­carboxylate [(1S*,2S*,5S*)-(±)-6]

A solution of (1S*,2S*,5S*)-(±)-**5** (200 mg), 10% Pd/C (40 mg) in EtOAc (15 mL) was stirred under H_2_ atmosphere at room temperature for 2 h. Then the Pd was filtered off through Celite and the filtrate was concentrated under reduced pressure. The crude product was purified on column chromatography on silica gel (*n*-hexane-EtOAc 1:1) and resulted (1S*,2S*,5S*)-(±)-**6** as a colourless oil; yield: 89%; (R_f_ = 0.4, *n*-hexane-EtOAc 1:1). ^1^H-NMR (400 MHz, CDCl_3_): δ = 1.24 (t, 3H, CH_3_, *J *= 7.10 Hz), 1.42 (s, 9H, *t*Bu), 1.57–2.00 (m, 6 H, CH_2_, 2.68–2.79 (m, 1H, H-1), 3.68–3.80 (m, 1H, H-2), 4.07–4.20 (m, 3H, OCH_2_ and H-5), 4.61 (brs, 1H, N-H). ^13^C-NMR (100 MHz, CDCl_3_): δ = 14.6, 27.2, 28.7, 31.6, 35.1, 44.6, 50.9, 61.0, 65.0, 79.7, 155.4, 174.3. 

### 3.7. Preparative-scale resolution of (1R*,2S*,5S*)-(±)-4

Compound (1R*,2S*,5S*)-(±)-**4 **(200 mg, 0.70 mmol) was dissolved in *t*-BuOMe (40 mL). CAL-B (1 g, 25 mg mL^-1^) and H_2_O (6.3 μL, 0.35 mmol) were added and the mixture was shaken in an incubator shaker at 60 °C for 10 days. The reaction was stopped by filtering off the enzyme at 43% conversion. The solvent was evaporated off, and the residue purified by column chromatography (*n*-hexane:EtOAc 1:3); (R_f_ = 0.6, *n*-hexane-EtOAc 1:3) to give (1*R*,2*S*,5*S*)-**4** [68 mg, 32%; [α]_D_^25^ = +34 (*c* 0.19 EtOH); m.p. 69–71 °C;*ee* = 74%]. The filtered-off enzyme was washed with distilled H_2_O (3 x 15 mL), than with MeOH (3 x 15 mL) and the combined solvents was evaporated off, yielding the mixture of mainly polymeric products and amino acid. ^1^H-NMR (400 MHz, CDCl_3_, 25 °C, TMS) data for (1*R*,2*S*,5*S*)-**4** were similar to those for (1R*,2S*,5S*)-(±)-**4**. Analysis: calculated for C_14_H_23_NO_5_ (285.34): C, 58.93; H, 8.12; N, 4.91; found: C, 58.80; H, 8.08; N, 4.92. 

### 3.8. Preparative-scale resolution of (1S*,2S*,5S*)-(±)-**5**

Following the procedure described above, the reaction of racemic (1S*,2S*,5S*)-(±)-**5 **(200 mg, 0.70 mmol) and H_2_O (6.3 μL, 0.35 mmol) in *t*-BuOMe (40 mL) on the presence of CAL-B (1 g, 25 mg mL^-1^) at 60 °C afforded after 10 days (1*S*,2*S*,5*S*)-**5** [64 mg, 32%; [α]_D_^25^ = +16 (*c* 0.165 EtOH); m.p. 100–102 °C;*ee* = 78%]. ^1^H-NMR (400 MHz, CDCl_3_, 25 °C, TMS) data for (1*S*,2*S*,5*S*)-**5** were similar to those for (1S*,2S*,5S*)-(±)-**5**. Analysis: calculated for C_14_H_23_NO_5_ (285.34): C, 58.93; H, 8.12; N, 4.91; found: C, 58.80; H, 8.08; N, 4.92. 

### 3.9. Preparative-scale resolution of (1S*,2S*,5S*)-(±)-**6**

Following the procedure described above, the reaction of racemic (1S*,2S*,5S*)-(±)-**6 **(200 mg, 0.69 mmol) and H_2_O (6.3 μL, 0.35 mmol) in *t*-BuOMe (40 mL) on the presence of CAL-B (1 g, 25 mg mL^-1^) at 60 °C afforded after 10 days (1*S*,2*S*,5*S*)-**6** [58 mg, 29%; [α]_D_^25^ = -4 (*c* 0.24 EtOH); yellowish oil;*ee* = 54%]. ^1^H-NMR (400 MHz, CDCl_3_, 25 °C, TMS) data for (1*S*,2*S*,5*S*)-**6** were similar to those for (1S*,2S*,5S*)-(±)-**6**. Analysis: calculated for C_14_H_25_NO_5_ (287.35): C, 58.52; H, 8.77; N, 4.87; found: C, 58.66; H, 8.62; N, 4.85. 

### 3.10. Preparative-scale resolution of (1S*,2S*,5R*)-(±)-**10**

Following the procedure described above, the reaction of racemic (1S*,2S*,5R*)-(±)-**10 **(200 mg, 0.70 mmol) and H_2_O (6.3 μL, 0.35 mmol) in *t*-BuOMe (40 mL) on the presence of CAL-B (1 g, 25 mg mL^-1^) at 60 °C afforded after 7 days (1*S*,2*S*,5*R*)-**10** [97 mg, 0.48%; [α]_D_^25^ = +100 (*c* 0.18 EtOH); m.p. 142–144 °C;*ee* = 90%]. ^1^H-NMR (400 MHz, CDCl_3_, 25 °C, TMS) data for (1*R*,2*R*,5*R*)-**5** were similar to those for (1S*,2S*,5S*)-(±)-**5**. ^1^H-NMR (400 MHz, CDCl_3_, 25 °C, TMS) data for (1*S*,2*S*,5*R*)-**10** were similar to those for (1S*,2S*,5R*)-(±)-**10**. Analysis: calculated for C_14_H_23_NO_5_ (285.34): C, 58.93; H, 8.12; N, 4.91; found: C, 58.91; H, 8.00; N, 4.97. 

## 4. Conclusions

Four hydroxy-substituted *β*-amino ester stereoisomers were resolved through a simple direct enzymatic method, involving CAL-B-catalyzed enantioselective hydrolysis in organic media. Moderate to good enantiomeric excess values (*ee* ≥ 52%) were obtained for the unreacted amino esters when the reactions were performed with 0.5 equiv. of added H_2_O, in *t-*BuOMe, at 60 ºC. Due to polymerization, the supposed *β*-amino acid enantiomers practically could not be isolated. 
